# Reconstruction of a Large Scalp Defect by the Sequential Use of Dermal Substitute, Self-Filling Osmotic Tissue Expander and Rotational Flap

**DOI:** 10.4103/0974-2077.69023

**Published:** 2010

**Authors:** Uwe Wollina, Yousef Bayyoud

**Affiliations:** *Department of Dermatology and Allergology, Section of Neurosurgery, Reconstructive and Hand Surgery, Hospital Dresden-Friedrichstadt, Academic Teaching Hospital of the Technical University of Dresden, Dresden, Germany*; 1 *Department of Trauma Surgery, Section of Neurosurgery, Reconstructive and Hand Surgery, Hospital Dresden-Friedrichstadt, Academic Teaching Hospital of the Technical University of Dresden, Dresden, Germany*

**Keywords:** Artificial dermal matrix, large scalp defects, self-filling osmotic expander

## Abstract

Large scalp defects pose a challenge for the surgeon. Here, we present a 31-year-old male patient with a soft tissue defect on the temple with exposed bone. To allow reconstruction, we placed a self-filling osmotic expander in the subgaleal pocket for 12 weeks. The final volume of the tissue expander was 300 mL. In the last step, a rotational flap was created after removal of the tissue expander from its pocket. Thereby, a tension-free suturing was possible. The post-surgical healing was uncomplicated. Osmotic tissue expanders are a valuable tool for the closure of large tissue defects without the necessity of repeated filling procedures.

## INTRODUCTION

Large scalp defects after accidents or tumour surgery represent a challenge for the surgeon. If the closure has to be combined with other techniques like cranioplasty, the planning of such a surgical procedure needs to address several concerns. Often, a step-by-step technique offers a greater flexibility and, potentially, a better outcome than single-step approaches. Interdisciplinary team work is most helpful.

Here, we report our experience with the reconstruction of a complex head injury after a severe traffic accident. We want to emphasize the opportunities offered by new technologies, such as artificial dermal matrices and osmotic tissue expanders, to solve such problems, with maximum benefits for the patient.

## CASE REPORT

**Figure 1:**
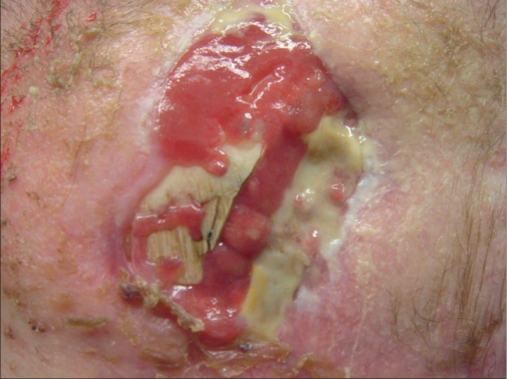
Large left temporal scalp defect, primary finding

A 31-year-old male patient presented with a skin defect on the left temple of about 6 cm in diameter after a traffic accident with severe cranial injury and fracture on the left frontal and temporal sites. Decompressive craniectomy was performed to treat intracranial hypertension refractory to medical therapy due to subdural haematoma. The soft tissue defect on his left temple resulted from traumatic descalping. Exposed bone could be seen at the margin [Figure 1]. Because the patient was in neurologic rehabilitation, primarily, a conservative approach was chosen. Cranioplasty with autologous bone graft or other reconstructive materials is generally considered as treatment options to repair the skull defect in such cases. In the present case, autologous bone was chosen for the planned cranioplasty.

## SURGICAL TECHNIQUE

The deep soft tissue defect on the left temple was covered by a dermal substitute to support healing and to prevent secondary infection, in particular of the exposed bone. We used MatriDerm^®^(Asklepios Medizintechnik, Gutach, Germany), a dermal template consisting of a native (non-cross-linked) collagen matrix supplemented by an elastin hydrolysate. It is available in sheets of 1 mm and 2 mm thickness and may be covered in a single-step procedure with immediate split-thickness skin grafting.[1,2]

In the present case, we wanted to improve wound closure without grafting because cranioplasty was necessary to close the large bone defect eventually [Figure 1]. Eight weeks later, a stable epidermal cover and a slightly atrophic scar developed. Continuation of neurologic rehabilitation was possible without the risk of secondary infection.

A cranioplasty with autologous bone was planned to close a large bone defect of the left os temporale. The bone had been preserved according to the procedure desribed by Osawa *et al*.,[3] with deep freezing and autoclaving before implantation. To reduce any post-operative risks for complications, the atrophic scar had to be removed (diameter 6 cm), which was located close to the bony defect. This would have left a large scalp defect. To obtain a tension-free closure of the scalp defect, we performed a two-step procedure.

In the first step, a rectangular osmotic tissue expander of 30 mL with a final volume of 300 mL (Osmed GmbH, Ilmenau, Germany) was employed [Figure 2a]. After a 3cm long incision, the expander was placed in an occipitotemporal subgaleal pocket. This location is ideal because of its relative avascularity, which allows an atraumatic undermining with gloved fingers. The procedure was performed under general anaesthesia [Figure 2b]. Antibiotic prophylaxis with cefuroxime 500mg, bid was prescribed.

**Figure 2a:**
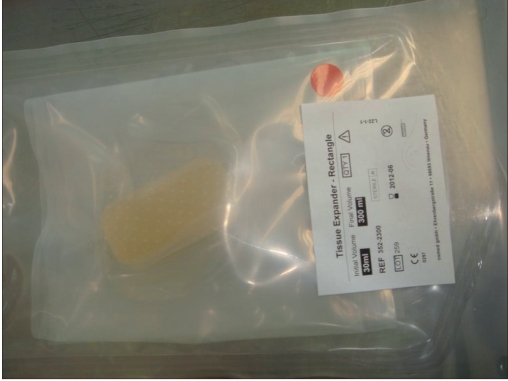
Osmotic tissue expander of 30 mL with a final expander volume of 300 mL before impanation

**Figure 2b:**
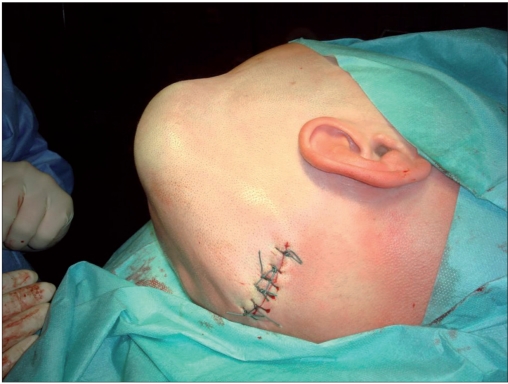
Placement of the tissue expander in a subgaleal pocket and wound closure

Wound healing of the small incision was rapid and without complications. The final expander volume was achieved after 12 weeks. The continuous expansion was well tolerated, without the need of any analgesics during the whole 12-week interval [Figure 2c].

**Figure 2c:**
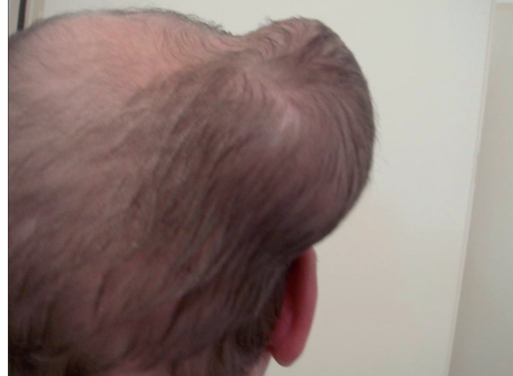
After 12 weeks, the final expander volume was achieved

In a second session, the skull defect was closed with autologous bone by the neurosurgeon (YB). Antibiotic prophylaxis was performed using ceftriaxone 2 g/day i.v. (Rocephin, Hoffmann-LaRoche).

Normal intracranial pressure (ICP) is below 10mm of Hg. It may increase as a result of surgery or other pathologies. When ICP increases above 20mm of Hg, it may damage the neurons and jeopardize cerebral perfusion. To prevent a cerebral oedema, a mannitol was given intravenously.[4]

The atrophic scar of about 6 cm in diameter was excised. After removal of osmotic tissue expander from its pocket [Figure 2d] and wide mobilization of the scalp, the scalp defect could be closed without tension by a single rotation flap [Figure 3a]. The flap was prepared by mobilization of the soft tissue from the galea with a monopolar electrosurgical knife.

**Figure 2d:**
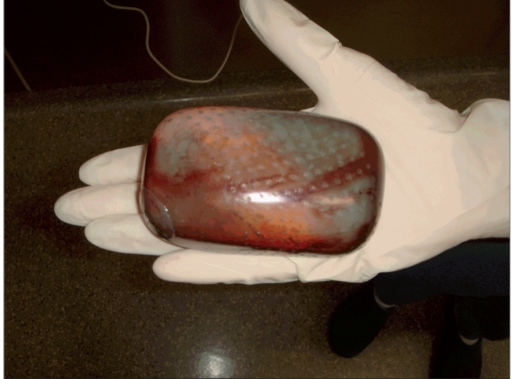
Removed osmotic tissue expander of 300 mL

**Figure 3a:**
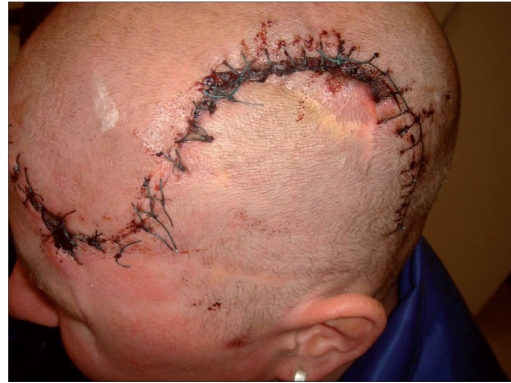
Seven days after surgery: tension-free sutures after complete removal of the atrophic scar on the left temple. The u-shaped sutures on the temporooccipital scalp cover the autologous bone implant

A post-traumatic lateral brow ptosis of the left side could be corrected by a triangular excision of skin with subcutaneous tissue and double-layer sutures adjacent to the excision line for the rotation flap.

Wound exudate and minor bleeding was collected by two Redon drainages, one for the skin covering the cranioplasty and the second for the scalp rotation flap. They were removed after 5 and 8 days respectively. The total amount of exudates collected was 150 mL.

The wound healing was uncomplicated. Sutures were removed within the next 2 weeks. The functional and aesthetic outcome was good [Figure 3b]. Scar lines would be covered by hair.

The healing of the autologous bone was controlled by magnetic resonance imaging. No significant bone resorption or bleeding was observed.

**Figure 3b:**
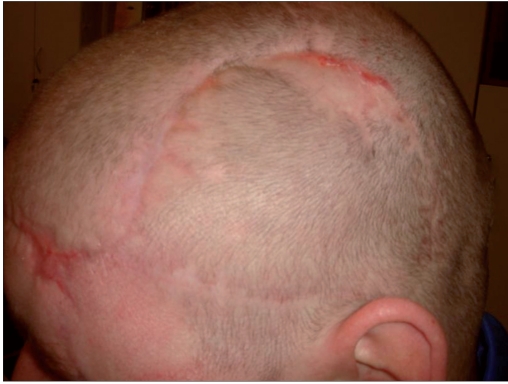
About 8 weeks later

## DISCUSSION

We were challenged by several problems in this case. The first problem was a soft tissue defect with exposed bone on the left temple. The defect was close to the skull defect after decompressive craniectomy. We preferred a conservative approach using an artificial dermal matrix, MatriDerm^®^. A variety of dermal templates are available on the market currently for this indication.[5,6] The templates can be combined with a mesh-graft. In this particular case, the skin graft would have been unsuitable for the final cranioplasty.

Cranioplasty was planned for the time after early neurologic rehabilitation. Cranioplasties to repair skull defects have an overall complication rate of about 16.4%. Patients with traumatic injuries who present earlier have a lower rate of complications than those who do not. Patients who received autologous bone graft placement have a statistically significant lower risk of post-operative infection (4.6 vs. 18.4%).[7] We decided to remove the atrophic scar close to the bony defect before cranioplasty to further reduce any risk of complication.

Size of defects in the scalp for which primary wound closure can be applied is limited. Cases with a scalp defect for which primary wound closure is difficult to perform are frequently seen. Various reconstructive techniques using hair-bearing scalp to manage unsightly scalp defects have been described, like extensive subgaleal undermining or galeal incisions, galeal-pericranial flaps and early intra-operative tissue expansion.[8]

Tissue expanders have been used since the ‘80s for large scalp defects and scars.[9–12] Careful expander selection and application can allow extensive reconstructions with adjacent tissues, which might otherwise be impossible. Complications are rare and usually avoidable with careful attention and technique.[9,13,14]

The traditional tissue expander needs a step-by-step inflation, often in a weekly or bi-weekly schedule. Complications include infection (6%), extrusion (3%), haematoma (2%), flap ischaemia (1%) and expander perforation after percutaneous stabbing (1%).[15] In breast surgery with tissue expanders, the total complication rate was 4%, with seroma and wound infection being the most common complications.[16] Anatomical region for the expander implantation and the total volume for tissue expansion are factors that influence the complication rate and outcome.[17]

Recently, osmotic tissue expanders are available which allow tissue expansion without the need of step-by-step filling procedures and repeated punctures. The material consists of a modified copolymer hydrogel of methylmethacrylate and N-vinyl-2-pyrrolidone covered by a silicone shell that allows an exact calculation of the final volume.[18–20] The expander is of the size of about 10% of the final volume and requires a short incision and a small pocket only. Insertion can easily be carried out with local anaesthesia.[21] The expansion-related swelling is well tolerated and the incidence of complications has been reported to be very low.[22,23]

In conclusion, osmotic tissue expanders can be used to treat larger scalp defects without the need for repeated punctures. The adverse effects are mild (mild post-surgical pain, limited pain during osmotic filling) and tolerable.

## CONUNDRUM KEYS

In this patient, cranioplasty was necessary to cover a large bone defect. To avoid the risk of secondary infection, the atrophic scar had to be removed.

The primary skin defect was safely treated by dermal collagen/elastin matrix without the need of skin transplantation.

An osmotic expander was used for tissue expansion, which provided the benefits of tissue expansion without the need for repeated filling procedures.

In a two-step procedure, both the bony defect and the large scalp defect after scar removal could be closed. Autologous bone material was used for the skull. A rotation flap was developed after tissue expander removal allowing a tension-free wound closure.

Large scalp defects need an individually tailored surgical approach. Interdisciplinary team work is most helpful. With the available techniques, even large defects and complicated situations can be treated.
